# SARS-CoV-2 isolation and propagation from Turkish COVID-19 patients

**DOI:** 10.3906/biy-2004-113

**Published:** 2020-06-21

**Authors:** Cihan TAŞTAN, Bulut YURTSEVER, Gözde SIR KARAKUŞ, Derya DİLEK KANÇAĞI, Sevda DEMİR, Selen ABANUZ, Utku SEYİS, Mülazim YILDIRIM, Recai KUZAY, Ömer ELİBOL, Serap ARBAK, Merve AÇIKEL ELMAS, Selçuk BİRDOĞAN, Osman Uğur SEZERMAN, Ayşe Sesin KOCAGÖZ, Koray YALÇIN, Ercüment OVALI

**Affiliations:** 1 Acıbadem Labcell Cellular Therapy Laboratory, İstanbul Turkey; 2 Genetic and Bioengineering Department, Faculty of Engineering, Yeditepe University, İstanbul Turkey; 3 Acıbadem Altunizade Hospital, İstanbul Turkey; 4 Department of Histology and Embryology, School of Medicine, Acıbadem Mehmet Ali Aydınlar University, İstanbul Turkey; 5 Electron Microscopy Laboratory, Acıbadem Mehmet Ali Aydınlar University, İstanbul Turkey; 6 Department of Biostatistics and Medical Informatics, School of Medicine, Acıbadem Mehmet Ali Aydınlar University, İstanbul Turkey; 7 Epigenetiks Genetics Bioinformatics Software Inc., İstanbul Turkey; 8 Infectious Disease Unit, Acıbadem Altunizade Hospital, İstanbul Turkey; 9 Pediatric Bone Marrow Transplantation Unit, Medical Park Göztepe Hospital, İstanbul Turkey

**Keywords:** COVID-19, SARS-CoV-2, coronavirus

## Abstract

The novel coronavirus pneumonia, which was named later as coronavirus disease 2019 (COVID-19), is caused by the severe acute respiratory syndrome coronavirus 2, namely SARS-CoV-2. It is a positive-strand RNA virus that is the seventh coronavirus known to infect humans. The COVID-19 outbreak presents enormous challenges for global health behind the pandemic outbreak. The first diagnosed patient in Turkey has been reported by the Republic of Turkey Ministry of Health on March 11, 2020. In May, over 150,000 cases in Turkey, and 5.5 million cases around the world have been declared. Due to the urgent need for a vaccine and antiviral drug, isolation of the virus is crucial. Here, we report 1 of the first isolation and characterization studies of SARS-CoV-2 from nasopharyngeal and oropharyngeal specimens of diagnosed patients in Turkey. This study provides an isolation and replication methodology,and cell culture tropism of the virus that will be available to the research communities.

## 1. Introduction

New coronaviruses are likely to occur periodically in humans, considering the high prevalence and wide distribution of coronaviruses, the large genetic diversity and frequent recombination of genomes, common interspecific infections, and rare outbreaks (Wong et al., 2015; Cui et al., 2019).The novel coronavirus pneumonia, which was named later as coronavirus disease 2019 (COVID-19), is caused by the severe acute respiratory syndrome coronavirus 2, namely SARS-CoV-2 (Holshue et al., 2020). It is a positive-strand RNA virus (family: Coronaviridae), showing high homology with SARS-CoV and bat coronavirus (Zheng 2020; Liu and Li, 2020). SARS-CoV-2 is the seventh coronavirus known to infect humans; SARS-CoV, MERS-CoV, and SARS-CoV-2 can cause severe disease, whereas HKU1, NL63, OC43, and 229E are associated with mild symptoms (Andersen et al., 2020). The novel SARS-CoV-2 is the virus behind the pandemic outbreak originating from China (Zhou et al., 2020) a large number of SARS-related coronaviruses (SARSr-CoVs and this recent coronavirus outbreak (COVID-19) presents enormous challenges for global health. The first diagnosed patient in Turkey was announced by the Ministry of Health on March 11, 2020. Since then, more than 150,000 diagnosed patients have been reported, showing the urgent need for vaccine studies and antiviral drug discoveries. Thus, it is important to isolate and propagate SARS-CoV-2 strains from Turkish COVID-19 patients. We isolated the virus from nasopharyngeal and oropharyngeal specimens. We characterized replication properties and cell culture tropism of SARS-CoV-2 in different cell lines including green monkey kidney cell line (Vero, ATCC CCL-81) and Madin-Darby bovine kidney cell line (MDBK, ATCC CCL-22). This study provides isolation and characterization methodology, which enables research communities to propagate the isolated virus in high-titer for vaccine development and antiviral drug screening.

## 2. Methods

### 2.1. Ethics approval and consent to participate

SARS-CoV-2 isolation and propagation were performed in Acıbadem Labcell Cellular Therapy Laboratory BSL-3 units within the scope of TÜSEB COVID-19 Virus Vaccine Development and TÜBİTAK-MAM Coronary Virus Injection and Drug Development Projects (33352965-517-E.98243). Each patient was informed and approved the patient information and consent form, which is named “Inactive COVID-19 Vaccine Production”.

### 2.2. Collection and transportation of specimen

Samples were collected from the nasopharyngeal and oropharyngeal cavity of COVID-19 positive diagnosed patients according to their real-time PCR analysis in Acıbadem Altunizade Hospital, İstanbul. Swabs were put into the transportation medium and transferred at 4 °C to Acıbadem Labcell Cellular Therapy Laboratory BSL-3 Units on the same day for analysis and propagation. Transfer medium contains DMEM high glucose (Thermo Fisher Scientific Inc., Waltham, MA, USA), 2% penicillin-streptomycin solution (Biological Industries, Beit HaEmek, Israel), and5 µg/mL amphotericin (Bristol Myers Squibb, New York, NY, USA). 

### 2.3. Virus isolation and propagation

The virus isolation and propagation process was started with the 96-well plate with a Vero cell line (CCl-81, ATCC) because of the low virus titer in inoculum samples (Harcourt et al. 2020). Firstly, Vero cells were trypsinized, centrifuged and suspended in virus media that is composed of DMEM high glucose (Thermo Fisher Scientific Inc.), with 2% fetal bovine serum (Thermo Fisher Scientific Inc.) and 1% penicillin-streptomycin-amphotericin (PSA) solution (Pan-Biotech GmbH, Aidenbach, Germany). Cells were seeded 2.5 × 104 into each well. Then, 100 µL of inoculum sample was added to the 1st line, and 50 µL of the virus media added to other wells of the plate. Serial dilution was done starting from the 1st line to 12th with 50 µL of virus medium (as 1/2 serial dilution) and incubated at 37 °C. Each day cytopathic effect was recorded under an inverted microscope (Leica Microsystems GmbH, Wetzlar, Germany) and after its detection; cells were scraped from the well and transferred to the 24-well plate with the Vero cell line in 25 × 104 concentrations per well. The cytopathic effect was observed and cells transferred to the T75 tissue culture flask. Virus replication and isolation was detected with observed cytopathic effect and virus isolate transferred to the T75 tissue culture flask prepared in 5 × 106 concentration. For higher propagation, virus isolate was transferred to the T300 tissue culture flask. At this point, the average number of days for cytopathic effect can be known as 3–4 days. 

### 2.4. Virus identification and characterization

The virus isolate were confirmed through loop-mediated isothermal amplification (LAMP) assay and transmission electron microscopy (TEM).

RNA isolation, reverse transcription, and LAMP assay: Total RNA isolations were carried using Direct-zol RNA Miniprep Kits (Zymo Research Corp., Tustin, CA, USA), and concentrations were determined using Qubit fluorometer with the Qubit RNA HS Assay (Thermo Fisher Scientific Inc.). cDNA synthesis was performed using random oligonucleotides. Relative flouresence units (RFU) was measured every 0.5 degree from 60 degrees to 95 degrees for the melting curve and this is repeated graphically by repeating twice. Melting curves are created by manufacturers preinstalled high-resolution melting settings of Bio-Rad CFX96. Two LAMP assay reactions were set-up in CFX96 (Bio-Rad Laboratories, Inc., Hercules, CA, USA) for each sample with SARS-CoV-2 LAMP primer mix and internal control LAMP primer mix (Primers designed by Epigenetiks Genetics Bioinformatics Software Inc., İstanbul, Turkey). EvaGreen fluorescent dye (Biotium, Inc., Fremont, CA, USA) was added to each reaction mixture for real-time LAMP detection. Melt curve analysis and 1% agarose gel electrophoresis were used for the evaluation of the results. Here, the virus gene encoding nucleocapsid (N) protein was used as a target gene in SARS-CoV-2 and actin as an internal control. Next, SARS-CoV-2 specific RT-PCR was performed with the LAMP-positive samples using Bosphore Novel Coronavirus (2019-nCoV) Detection Kit (Anatolia Geneworks, Anatolia Diagnostics and Biotechnology Products Inc., İstanbul, Turkey) along with Orf1ab and E gene primers. The RT-PCR analysis was performed in Roche LightCycler 96.

Transmission electron microscopy (TEM): Infected Vero cells were scraped from the flask and centrifuged at 300XG for 10 min. The cell pellet was rinsed with 0.1 M phosphate buffer saline (Thermo Fisher Scientific Inc.) centrifuged at 300XG for 10 min. Pellet was fixed 2.5% glutaraldehyde in PBS (0.1 M, pH 7.2) for 2 h, and then postfixed with 1% osmium tetroxide in PBS (0.1 M, pH 7.2) for 1 h at room temperature. Pellet was en bloc stained with 4% uranyl acetate, dehydrated with increasing concentrations of ethyl alcohol, and embedded in Epon 812 resin. Ultrathin sections of 600 angstroms were stained with 4% uranyl acetate and lead citrate. Ultrathin sections were evaluated under a transmission electron microscope (Thermo Fisher Scientific Inc.-Talos L120C) and photographed.

CPE assay: CPE test was performed based on general procedure with small modifications as following. Vero CCL-81 cells and MDBK CCL-22 cells were seeded for a 6-well plate (1 × 105 cells/well) and left for 24 h incubation at 37 °C. After cell incubation period, virus sample was serially diluted log10 for virus CPE titration assay. Cells were washed with cell media (DMEM High, Thermo Fisher Scientific Inc.) that contain only 1% PSA (Pan-Biotech GmbH). Virus dilutions were inoculated to cells in 1 mL volume, left for the 1-h incubation, and plates were shaken gently in 20 min. In this period, also cell culture medium that contains 2% agarose and DMEM cell culture media (2% FBS and 1% PSA) in 1:1 volume ratio were prepared and put into the 56 °C water bath for 30 min. After virus inoculation, nonadsorbed viruses were removed and 1 mL warm CPE medium added on infected cells. Plates were left under the hood for 15 min without a cap and then put into 37 °C incubator for 7–10 days. For the determination of the CPE firstly cells were fixated with 10% of paraformaldehyde for 1 h. Agarose layers were removed from the well and then 500 µL of undiluted crystal violet solution was added to each well and incubated on a shaker for 5–10 min. Crystal violet was discarded and plates were washed with tap water without disrupting cell monolayer until the water was clear. Plates were left on a paper towel till dry. CPE were imaged under an inverted microscope.

### 2.5. Immunological response

Inactivation of virus: Infected Vero cells were scraped from the flask and centrifuged at 1000 rpm for 10 min. The cell pellet was rinsed with 0.1 M phosphate buffer saline (Thermo Fisher Scientific Inc.) centrifuged at 1000 rpm for 10 min. Pellet was fixed 2.5% glutaraldehyde (GA) in 0.1 M phosphate buffer saline (PBS) (pH 7.4) for at least 2 h at room temperature (Harcourt et al. 2020). Virus solution (15 mL) was added to Amicon Ultra 50,000 KDa NMWL and centrifuge at 4,000XG for 15 min to wash from excess GA. For washing sample was diluted with 1mL of PBS and centrifuge for 15 min at 4,000XG. The sample was diluted with 1ml of PBS and centrifuge for 3 min at 4,000XG. The concentrator was transferred to a clean tube and centrifuge at 1,000XG for 2 min to collect the concentrated sample. The sample was diluted in serum physiologic.

PBMC culturing: Three healthy adult blood was obtained from Acıbadem Labcell Cellular Therapy Laboratory. Following the isolation of peripheral mononuclear cells (PBMC) by overlaying blood on Ficoll-Paque Plus (GE Healthcare Bio-Sciences AB, Uppsala, Sweden), the serially diluted GA-inactivated SARS-CoV-2 samples were incubated with the PBMCs for 48 h in T cell medium (6% Human AB Serum and 1% Pen/Strep, TexMACS Medium). ImmunoCult Human CD3/CD28 T Cell Activator (STEMCELL Technologies Inc., Cambridge, MA, USA) was used to stimulate the PBMCs as a positive control for immunological activation.

Flow cytometry: Immune cell subtypes and their activation levels were determined by Miltenyi MACS Quant flow cytometry analysis using aCD3-PE, aCD19-PE.cy7, aCD56-FITC, aCD4-Viogreen, aCD8-Vioblue, aCD25-APC and aCD107a-PE.cy5.5 (Miltenyi Biotec GmbH, Bergisch Gladbach, Germany). 

Interferon-gamma ELISA: Three human PBMC’s were added to different inactivated SARS-CoV-2 virus diluted to 5 different dilutions. After 48 h, supernatants were collected and cytokine levels assed with Human IFNγ ELISA Kit (Thermo Fisher Scientific ImmunoDiagnostics/Phadia Austria GmbH, Vienna, Austria) according to the manufacturer’s instructions. The 50 µL supernatants [test samples, positive control (ImmunoCult-activated) and negative control] were added into 450 µL sample diluents (TexMACS) in tubes (1:10 serial dilution) and 50µL diluents were added into wells. ELISA plate was measured at 450 nm, and 550 nm using a Microplate Reader (BMG Labtech GmbH, Offenburg, Germany).

### 2.6. Statistics

In the bar graphs, t-tests were performed using SPSS Statistics software. No outliers were excluded in any of the statistical tests and each data point represents an independent measurement with three healthy adult donor PBMCs. Bar plots report the mean and standard deviation or the standard error of the mean. Threshold of significance for all tests was set at P < 0.05. 

## 3. Results

### 3.1. Propagation and genetic confirmation of SARS-CoV-2 collected from COVID-19 patient

**Figure 1:**
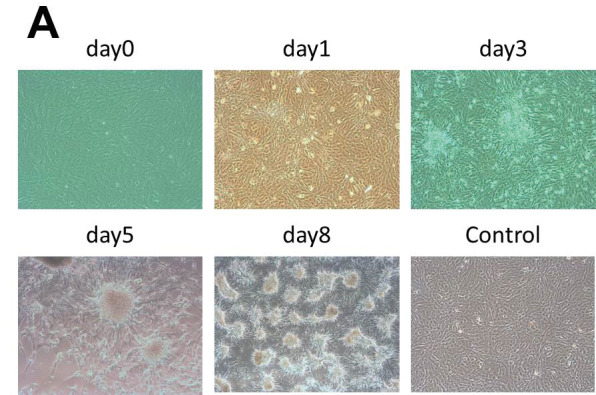

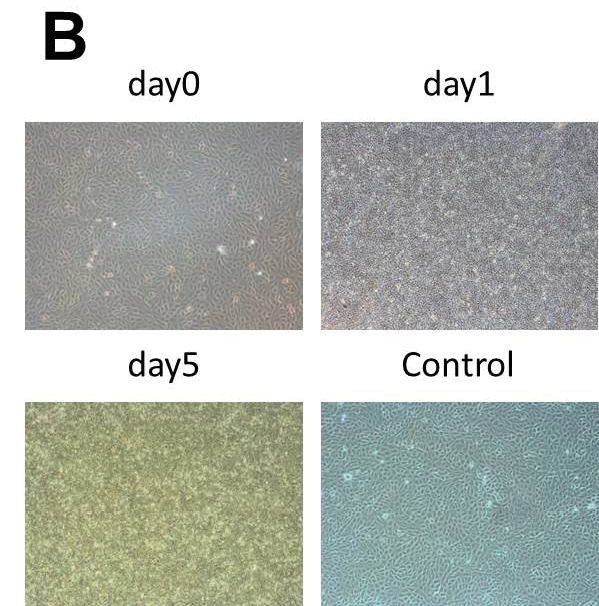

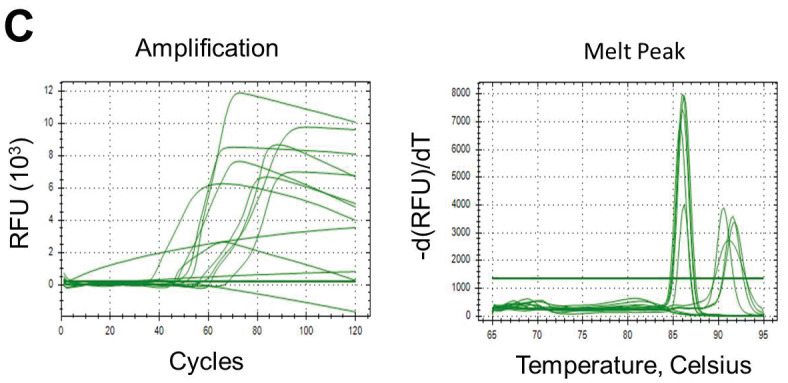

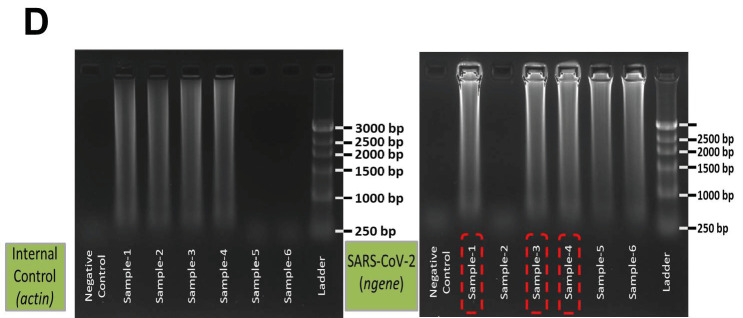

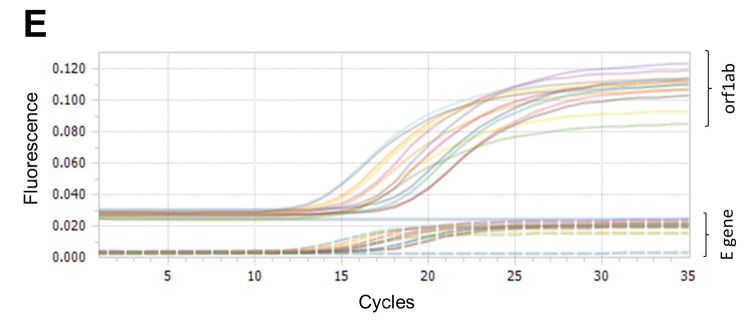
SARS-CoV-2 propagation and genetic confirmation.A. Images of virus propagation in Vero cell line with respect to the control Vero cell line without virus inoculation. B. Images of virus propagation in MDBK cell line along with the control MDBK cell line that was not infected with virus. C. Graphics obtained from a representative LAMP assay carried on real time monitoring system (Bio-Rad CFX96 Touch) with detection of EvaGreen fluorescent dye. Amplification curve (left) and melt peak (right) of LAMP assay carried with internal control primers (actin) and SARS-CoV-2 primers. D. Agarose gel electrophoresis of the amplicons obtained after LAMP assay shown in C. Products were run on 1% agarose gel, and stained with SYBR Safe (Thermo Fisher Scientific Inc.). Sample 1 to 6 are swap samples collected from COVID-19 patients. According to the results, sample 1, 3, and 4 were determined as SARS-CoV-2 positive. E.RT-PCR amplification curves of the LAMP-positive SARS-CoV-2 samples that were specifically amplified with SARS-CoV-2 Orf1ab and E gene primers.

The SARS-CoV-2 including swab specimens collected from COVID-19 diagnosed patients were quickly transferred on the same day to the laboratory and incubated with Vero cell in 96-well plate as mentioned in the method. Because of low titer viruses, initial small size culture led to easy recovery. Upon observation of the first cytopathic effect, supernatant and the cells were transferred to a 24-well plate with suspension form of Vero cell. Through 1 week, the propagation of the virus was followed and increasing cytopathic effects were recorded with respect to the control Vero cell line without virus inoculum (Figure 1A). As bovine coronavirus (B-CoV) is incubated with a bovine kidney cell line (MDBK) (Matsumoto et al., 2005), we wanted to test propagation of the SARS-CoV-2 with this cell line. It showed a cytopathic effect faster than the Vero cell line compared with control MDBK cell line without virus inoculation (Figure 1B). To verify the presence of the SARS-CoV-2 virus in the propagated samples, the LAMP assay was conducted. After isolation of total RNA and conversion to cDNA, LAMP assay was carried on a real-time monitoring system with the detection of signals coming from EvaGreen dye bound to amplicons (Figure 1C). For verification of the LAMP assay products, melt curve analysis was added to the LAMP assay program, and agarose gel electrophoresis was carried (Figures 1C and 1D). Samples giving internal control (*actin*) and SARS-Cov-2 amplicons were evaluated as positive (Figure 1D). Afterward, RT-PCR was performed with the LAMP-positive samples using SARS-CoV-2 Orf1ab and E gene primers (Figure 1E). This data further confirmed the SARS-CoV-2 isolation and propagation from 3 out of 6 patient isolates.

### 3.2. Transmission electron microscopy imaging and cytopathic effect (CPE) assay of SARS-CoV-2

**Figure 2:**
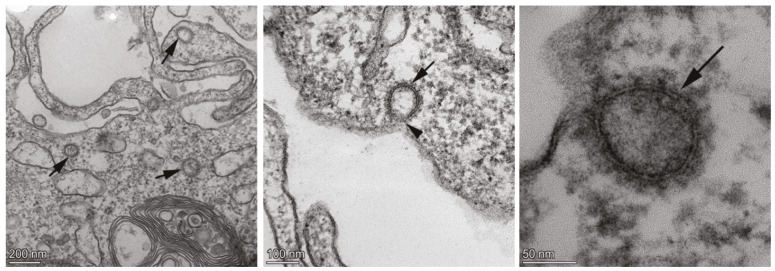
Transmission electron microscopy images of SARS-CoV-2 propagation in Vero cell line.Arrows show the virus in the images taken at different magnifications (200–100–50 nm).

**Figure 3:**
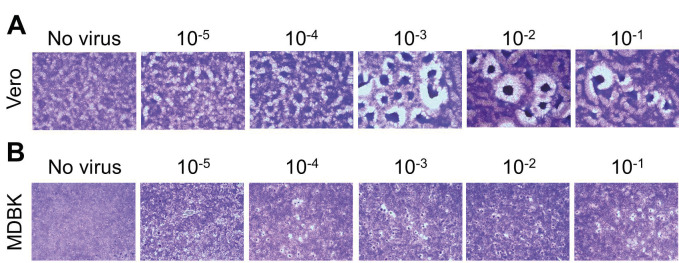
CPE assay of SARS-CoV-2 in A.Vero and B.MDBK cell lines with different virus titers.Images are taken in 6th day of the assays.

Ultrastructural investigation of the SARS-CoV-2 was performed with transmission electron microscopy (TEM). TEM analysis showed that SARS-CoV-2 was located within the cell line samples (Figure 2). The entry of SARS-CoV-2 into the cell through caveolar structures has been recognized in the sections. Ultrastructural organization of the spike protein of SARS-CoV-2 was prominently detected in the sections. Based on the ultrastructural data, the propagation of the virus within the cell line has been concluded (Figure 2). CPE assay is another way to show virus infectivity (Guallart, 2015). Therefore, this assay was performed on Vero and MDBK cell lines for isolated and propagated virus. Although the literature (Harcourt et al. 2020) suggests that CPE formation was determined in 72 h, the CPE were observed in our experiments after 6 days in both cell lines (Figure 3). The results confirmed our methodological approach for the isolation and propagation of SARS-CoV-2.

### 3.3. Immunological response of inactivated virus

**Figure 4:**
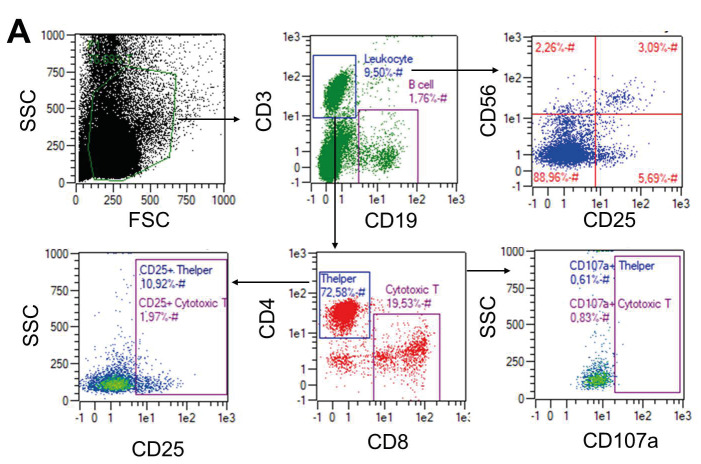

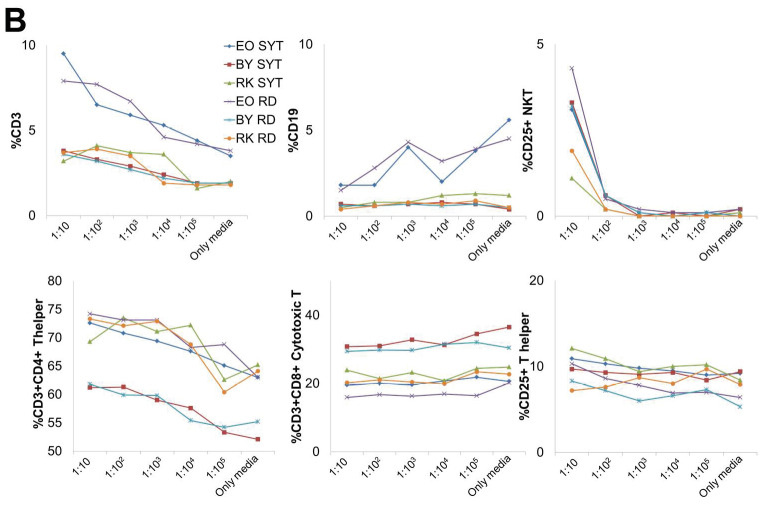

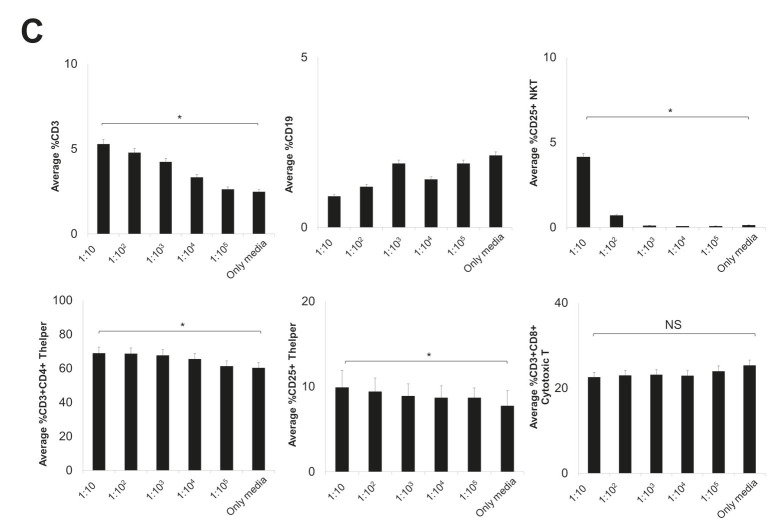
Stimulated immune response by GA-inactivated SARS-CoV-2.A.Flow cytometry plots that show immune cell subtypes (CD19+ B, CD3+ CD4+ T helper, CD3+ CD8+ cytotoxic T, and CD3+ CD56+ Natural Killer T (NKT) cells) and their activation (with aCD25 and aCD107a). EO, BY and RK are healthy human PBMCs. Also, SYT and RD are propagated SARS-CoV-2 samples from 2 COVID-19 patients. Graphs that show B.frequencies (linear graphs) and C.averages (bar graphs) of the immune cell subtypes and their activation from 3 healthy donor peripheral blood mononuclear cells incubated with 2 different SARS-CoV-2 samples. P < 0.05, and NS: Not significant.

**Figure 5:**
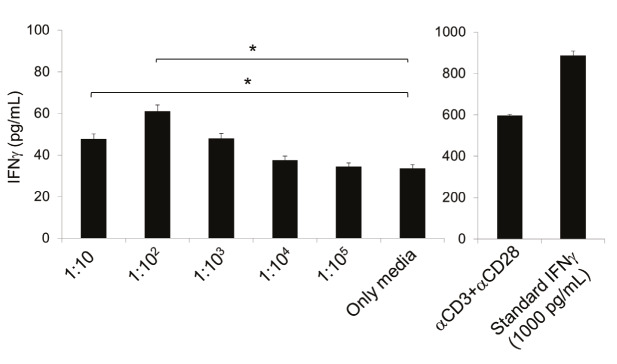
IFNg secretion capacity of the stimulated PBMCs by SARS-CoV-2 in a dose-dependent manner.The graphs show IFNg secretion (pg/mL) upon 48 h incubation of the PBMCs with the virus (left graph) or with aCD3+aCD28 ImmunoCult (right graph). P < 0.05.

PBMCs from healthy donors are a useful tool for assessment of host response upon pathogen infection. The cells can be stimulated by encountering SARS-CoV and recruit immune cell subtypes such as natural killer T (NKT), T, and B cells to the site of inflammation to give an early adaptive immune response (He et al., 2006). Therefore, we performed immunological assays to show the impact of GA-inactivated SARS-CoV-2 on frequencies and activation capacities of the cell subtypes. We incubated healthy adult PBMCs with the inactivated virus in a dose-dependent manner for 48 h and assessed their proportions and expression level of activation markers (CD25 and CD107a) using flow cytometry as shown in Figure 4A. We determined an increase in the frequency of CD3+ T cells, especially CD3+ CD4+ T helper cell, except CD3+ CD8+ cytotoxic T cells at the highest concentration of the virus (Figures 4B and 4C**)**. On the other hand, CD19+ B cell proportion has decreased upon increasing the virus concentration, suggesting activation of the immune cells (Figures 4B and 4C). Afterward, we assessed activation of the T cells and NKT cells, and we determined a significant upregulation of CD25 on CD3+ CD56+ NK T cells and an increase of CD25 level in CD3+ CD4+ T helper cell but not in CD3+ CD8+ cytotoxic T cells (Figures 4B and 4C). However, we could not detect the expression of a degranulation marker (CD107a) on the cells (data not shown). These results led us to determine another immunological stimulus marker (IFNg secretion) using collected supernatant of the samples after 48 h. We determined a significant increase inIFNg secretion (Figure 5) using ELISA, which confirm the occurrence of the immune response with chemically inactivated SARS-CoV-2. 

## 4. Discussion

Coronaviruses are enveloped RNA viruses that are widely distributed among humans, other mammals, birds, which cause respiratory, enteric, hepatic, and neurological diseases (Weiss and Leibowitz, 2011; Masters and Perlman, 2013). After the first outbreaks of unexplained pneumonia in Wuhan, China, a new coronavirus was detected as a disease-causing agent in late 2019 and January 2020 (Paraskevis et al., 2020) shows discordant clustering with the Bat_SARS-like coronavirus sequences. In April 2020, above 2 million cases have been reported from 26 countries, including China (Hui et al., 2020). In May 2020, above 5.5 million cases have been recorded. The emergence of coronaviruses at regular intervals poses an important threat to human health and the economy of countries. Ironically, even after a decade of research on the coronavirus, there are no licensed vaccines or therapeutic agents to treat coronavirus infection. This emphasizes the urgent need to develop effective vaccines to prevent future outbreaks (Li et al., 2020).

In the process of specimen collection from patients and transfer to the laboratory, the process should be started and virus cultured immediately because lots of viruses became inactivated. Therefore, we tested several transfer solutions to safe infectious SARS-CoV-2 during transportation and we decided FBS-free media is the best for protecting the infectivity of the virus. Also, before culturing the cells with the specimens, we wash the cells with FBS free media to remove the excess of fetal bovine serum (Baer and Kehn-Hall, 2014). Then, to propagate the virus, cells were suspended with a virus media including 2% FBS, which enabled much easier propagation of the virus than the media including 10% FBS (Honda-Okubo et al., 2015). Afterward, we tested Vero and MDBK cell lines to observe cytopathic effects and it was determined that the spreading of SARS-CoV-2 occurred in MDBK cells faster than in Vero cells. Also, the cytopathic effect in the Vero cell line was observed as a clump form while in the MDBK cell line as a single cell base. CPE assay is a reliable way to show an infectious form of viruses. Therefore, in this study, we performed CPE assay to see the infectivity of SARS-CoV-2. During the CPE assay processes, we observed that the monolayer form of Vero cells behaves more sensitive than that of MDBK cells, which makes difficulties in the setup of the assay. Because of low titer viruses, cytopathic effect in Vero cells was determined later than as reported (Harcourt et al. 2020). Through 1 week, the propagation of the virus was followed and increasing cytopathic effects were recorded with respect to the control Vero cell line without virus inoculum. Since control Vero cells were comparatively confluent and intact with respect to the cells inoculated with the virus, this CPE is thought to be due to increase in virus infection. In the assay process, 90%–100% confluent Vero cells can be easily detached from the plate while virus inoculation and covering with the agarose, that may affect the determination of real virus-dependent CPE formation; however, CPE assay with the MDBK cell line was easy to perform, leading to safer result in CPE assay. Therefore, our study suggests that the MDBK cell line is efficient for CPE assay for virus titration and antiviral drug screening. Furthermore, studies reported that CPE formation time for SARS-CoV-2 was 72 h, however; in our study, CPE formation was observed on the 6th day of the infection, probably because of the virus low titer. Thus, we suggest waiting for 10 days postinfection to determine the titer of the virus by determining CPE.

Peripheral blood mononuclear cells are used to test immune response stimulated by pathogens. To determine immune cell activation through the proteins of inactivated SARS-CoV-2, we incubated PBMCs with a chemically-inactivated virus. The study shows us that T cells and NKT cells can be stimulated with the virus and can be assessed significantly in vitro setup. We also observed a decrease in B cell population in higher concentrations of the inoculums; probably activated and proliferated T cells interfere with B cell proliferation (Stohl and Mayer, 1987). To further confirm the activation, we determined IFNg secretion from the stimulated cells in PBMCs. These preliminary results and other studies showing that SARS-CoV antigens or synthetic peptides stimulated CD3+ T cells in human PBMC in vitro (Libraty et al., 2007; Li et al., 2008) aiming to identify the immune correlates of protection. 

To conclude, this study can be referenced for researchers who aimed to isolate and propagate SARS-CoV-2 to work in molecular biology studies including genomics, proteomics, and gene editings like CRISPR, antisense peptide, and small interference RNA (siRNA) therapies. 

## Acknowledgement/Disclaimer

The authors would like to thank Prof. Fikrettin Şahin and Prof. Dilek Telci from Genetic and Bioengineering Department, Faculty of Engineering, Yeditepe University, İstanbul, Turkey for their comments and their insightful suggestions for the study. All funding in the work was supported by Acıbadem Healthcare Group, İstanbul, Turkey.
